# NF-κB-direct activation of microRNAs with repressive effects on monocyte-specific genes is critical for osteoclast differentiation

**DOI:** 10.1186/s13059-014-0561-5

**Published:** 2015-01-05

**Authors:** Lorenzo de la Rica, Antonio García-Gómez, Natalia R Comet, Javier Rodríguez-Ubreva, Laura Ciudad, Roser Vento-Tormo, Carlos Company, Damiana Álvarez-Errico, Mireia García, Carmen Gómez-Vaquero, Esteban Ballestar

**Affiliations:** Chromatin and Disease Group, Cancer Epigenetics and Biology Programme (PEBC), Bellvitge Biomedical Research Institute (IDIBELL), 08908 L’Hospitalet de Llobregat, Barcelona, Spain; Present address: Barts and The London School of Medicine and Dentistry, Centre for Neuroscience & Trauma, Blizard Institute, Queen Mary University of London, 4 Newark Street, London, E1 2AT UK; Rheumatology Service, Bellvitge University Hospital (HUB), 08908 L’Hospitalet de Llobregat, Barcelona, Spain

## Abstract

**Background:**

Monocyte-to-osteoclast conversion is a unique terminal differentiation process that is exacerbated in rheumatoid arthritis and bone metastasis. The mechanisms implicated in upregulating osteoclast-specific genes involve transcription factors, epigenetic regulators and microRNAs (miRNAs). It is less well known how downregulation of osteoclast-inappropriate genes is achieved.

**Results:**

In this study, analysis of miRNA expression changes in osteoclast differentiation from human primary monocytes revealed the rapid upregulation of two miRNA clusters, miR-212/132 and miR-99b/let-7e/125a. We demonstrate that they negatively target monocyte-specific and immunomodulatory genes like *TNFAIP3*, *IGF1R* and *IL15*. Depletion of these miRNAs inhibits osteoclast differentiation and upregulates their targets. These miRNAs are also upregulated in other inflammatory monocytic differentiation processes. Most importantly, we demonstrate for the first time the direct involvement of Nuclear Factor kappa B (NF-κB) in the regulation of these miRNAs, as well as with their targets, whereby NF-κB p65 binds the promoters of these two miRNA clusters and NF-κB inhibition or depletion results in impaired upregulation of their expression.

**Conclusions:**

Our results reveal the direct involvement of NF-κB in shutting down certain monocyte-specific genes, including some anti-inflammatory activities, through a miRNA-dependent mechanism for proper osteoclast differentiation.

**Electronic supplementary material:**

The online version of this article (doi:10.1186/s13059-014-0561-5) contains supplementary material, which is available to authorized users.

## Background

The successful generation of differentiated cell types from their progenitors depends on the highly coordinated regulation of gene expression by transcription factors, epigenetic enzymes, and small non-coding RNAs, of which microRNAs (miRNAs) are the best studied. These regulate gene expression through sequence complementarity with their target mRNAs by mediating their decay or interfering with their translation [1]. miRNAs are known to have a major role in cell differentiation. However, their specific contribution in terminal differentiation processes remains poorly understood.

One such process is monocyte (MO)-to-osteoclast (OC) differentiation. OCs are giant, multinucleated cells that degrade bone [2] and differentiate from monocytic progenitors under inflammatory conditions. Their deregulation is associated with several diseases, either through deficient function that results in osteopetrosis [3] or aberrant hyperactivation that gives rise to generalized bone loss in osteoporosis [4] and rheumatoid arthritis [5]. Moreover, OCs cause bone complications in multiple myeloma [6] and in cancer metastasis, including prostate and breast cancers [7]. OCs differentiate from MO/macrophage progenitors [8] after macrophage colony-stimulating factor (M-CSF) [9] and receptor activator of nuclear factor kappa-B ligand (RANKL) [10] stimulation. *In vitro* generation of OCs from peripheral blood MOs allows differentiation to be studied in this model, since isolation of primary bone OCs can otherwise be challenging [11]. During osteoclastogenesis, progenitor cells fuse, reorganize their cytoskeleton [12] and activate the gene expression profile necessary for bone destruction. Several signaling pathways activate nuclear factor-kappa B (NF-κB), mitogen-activated protein kinase (MAPK) and c-Jun [13], which coordinately turn on NFATc1 [14], the osteoclastogenesis master transcription factor. NFATc1 acts in conjunction with PU.1 and MITF [15], activating OC-specific genes such as those encoding tartrate-resistant acid phosphatase (*TRAP* or *ACP5*) [16], cathepsin K (*CTSK*) [17], dendritic cell-specific transmembrane protein (*DC-STAMP* or *TM7SF4*) [18], matrix metallopeptidase 9 (*MMP9*) [19] and carbonic anhydrase 2 (*CA2*). Most importantly, other genes like *CX3CR1*, a MO-specific gene, and *TNFAIP3*, a deubiquitinating protease that mediates TRAF6 degradation and impairs NF-κB activation [20], need to be silenced during OC differentiation. It is not well understood how the silencing program is established during MO-to-OC differentiation. The importance of miRNAs in OC differentiation has been established through the observation that knock-out models for the miRNA processing machinery impair OC formation and reduced expression of TRAP and NFATc1 [21]. In addition, silencing of miRNAs, such as miR-29b [22] and miR-124 [23], is essential for the upregulation of pro-osteoclastic genes.

In this study, we investigated the role of miRNAs in establishing and maintaining a repressive program during OC differentiation. To this end, we performed high-throughput miRNA expression profiling of human peripheral blood MOs before and 2 and 20 days after stimulation with RANKL and M-CSF. We identified different dynamics in miRNA expression changes. Two miRNA clusters, miR-212/132 and miR- 99b/let-7e/125a, are highly upregulated during the early stages of osteoclastogenesis. Functional analysis of these miRNAs revealed that their depletion impairs proper OC differentiation. Interestingly, these miRNAs target MO-specific and anti-inflammatory genes that are downregulated during differentiation, such as *TNFAIP3*, *IGF1R*, *THBS1*, *ITGA4*, *IL15* and *PTGS2*. We investigated the potential involvement of the NF-κB transcription factor in the upregulation of these miRNAs. We demonstrated the direct association of p65 NF-κB with the transcription start site (TSS) of these miRNA clusters. Most importantly, we found that the pharmacological inhibition of the p65 subunit of NF-κB or its depletion results in impaired overexpression of these miRNAs and affects the downregulation of their targets. Our results demonstrate the direct relationship between p65 NF-κB and miRNA-mediated repression of several MO-specific and anti-inflammatory genes that is key for proper osteoclastogenesis and reveal novel potential pathways for therapeutic intervention in the treatment of bone complications in diseases such as rheumatoid arthritis and bone metastases.

## Results

### The miRNA expression profile changes drastically during osteoclastogenesis

**Figure 1:**
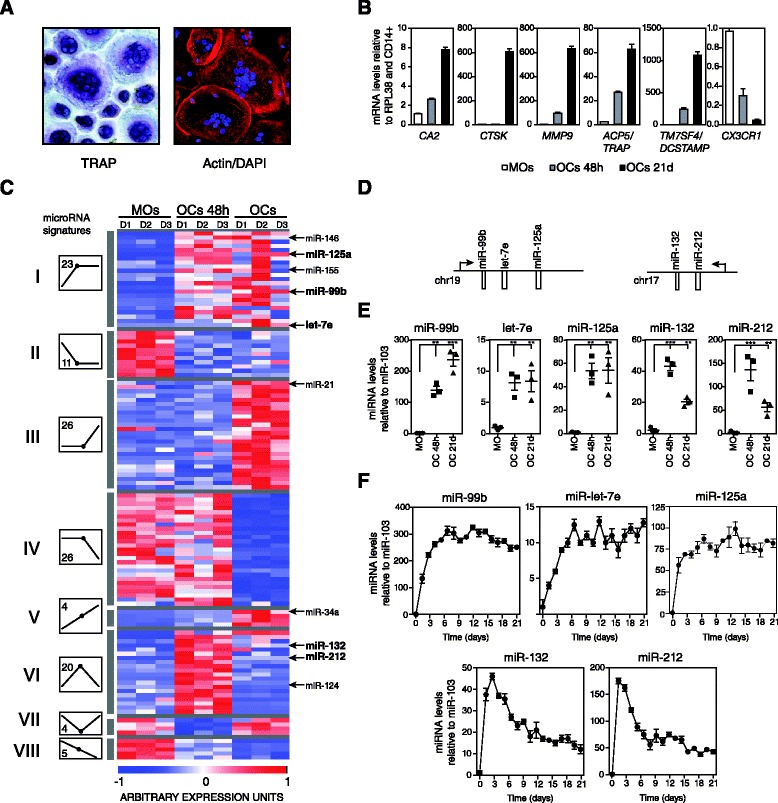
**MicroRNA expression profiling during monocyte-to-osteoclast differentiation. (A)** Validation of the presence of OCs by TRAP and phalloidin staining, showing the presence of TRAP activity/multiple nuclei and the actin ring, respectively. **(B)** Molecular characterization of OC differentiation. Several OC markers are upregulated (*CA2*, *CTSK*, *MMP9*, *ACP5*/*TRAP*, and *TM7SF4*/*DCSTAMP*), and the MO marker *CX3CR1* is silenced. Data for MOs, MOs 48 h after M-CSF and RANKL treatment and OCs at 21 days are presented. RPL38 gene expression levels were used for normalization. Error bars correspond to the standard deviation of three individual measurements. **(C)** Heatmap showing expression array data from the miRNA expression screening. miRNAs were subdivided into eight groups (I to VIII) according to their expression profile (diagram); the number of miRNAs in each group is indicated inside the expression dynamics diagram. Scale shown at the bottom, whereby normalized expression units ranges from -1 (blue) to +1 (red). **(D)** Representation of the genomic distribution of miR-99b/125a/let7e and miR-132/212 clusters, including the TSS (indicated with an arrow). **(E)** Validation of array data by quantitative PCR in independent biological replicates. Analysis in MOs, MOs incubated 48 h with RANKL/M-CSF and fully differentiated OCs. Data normalized with respect to miR-103. **(F)** Expression dynamics of the indicated miRNAs during OC differentiation, also normalized with respect to miR-103.

To determine the dynamics of miRNA expression during human osteoclastogenesis, we first generated three sets of matching samples corresponding to peripheral blood MOs (CD14+ cells), MOs 48 hours after RANKL/M-CSF treatment, and mature OCs obtained from the same sets of MOs, 21 days after RANKL/M-CSF stimulation. The quality of the OCs was confirmed microscopically by the presence of three or more nuclei in TRAP-positive cells and the formation of the actin ring (Figure 1A). At the molecular level, we confirmed the upregulation of osteoclastic markers (*CA2*, *CTSK*, *MMP9*, *ACP5*/*TRACP* and *TM7SF4/DCSTAMP*) and the silencing of the MO-specific gene *CX3CR1* (Figure 1B). We then performed miRNA expression profiling during the differentiation of MOs to OCs using the three sets of samples. Statistical analysis of the combined expression data from three biological replicates showed 115 miRNAs that were differentially expressed at one or more of the times analyzed (Figure 1C; Additional file 1). miRNAs displayed different expression profiles over time that enabled them to be classified into eight groups (Figure 1C) according to the combination of upregulation or downregulation at the initial or late stages of OC differentiation. Of particular interest were the miRNAs whose expression increased rapidly in the initial stages (groups I, V and VI; Figure 1C), regardless of their subsequent changes over time. miRNAs that become upregulated immediately after M-CSF and RANKL stimulation are potentially more important for the differentiation process than for the function of fully differentiated OCs. miRNAs within two clusters ranked top in terms of the coefficient of change and relative expression levels, specifically miR-99b/let-7e/125a (group I, average fold change = 49.4 between MOs and 48 h post-MCSF/RANKL stimulation) and miR-212/132 (group VI, average fold change = 50.57 between MOs and 48 h post-MCSF/RANKL stimulation) (Figure 1D). Several other activated miRNAs identified in our analysis have already been described in human and mouse experiments concerning OC differentiation (Figure 1C) like miR-124, a negative regulator of NFATc1 expression [23], and miR-155, also upregulated in bone marrow macrophage-derived OCs [24,25].

We confirmed the overexpression of all the miRNAs within the miR-99b/let-7e/125a and miR-212/132 clusters using quantitative RT-PCR (qRT-PCR) (Figure 1E). This analysis also confirmed that individual miRNAs from each of the two clusters do not reach the same expression levels. For example, miR-99b and miR-125a levels are increased by 300-fold and 100-fold respectively, whereas miR-let-7e induction is only increased by 10- to 12-fold. This strongly suggests that miRNAs in these clusters are regulated not only transcriptionally but also post-transcriptionally during MO-to-OC differentiation, as it has previously been observed for other miRNAs in other differentiation programs [26]. To refine the expression dynamics of these miRNAs during the differentiation process further, we generated a time course of osteoclastogenesis from three different healthy donors, and checked the miRNA levels at several times during the entire differentiation process. The two clusters showed different dynamics when we analyzed their expression levels over time. Specifically, after RANKL/M-CSF stimulation, the miR-99b/let-7e/125a cluster miRNAs underwent rapid overexpression during the first four days and the levels remained stably high until day 21 (Figure 1F, top). In contrast, miR-212/132 cluster miRNAs peaked at day 3, displaying an increase of around 50-fold (miR132) to 170-fold (miR-212), followed by an approximately 5-fold drop (Figure 1F, bottom). This suggests that the functions of miR-132 and miR-212 are involved in the early events of osteoclastogenesis, since their expression levels are tightly regulated and constrained to the first four days of differentiation.

### Inhibition of miRNAs within the miR-99b/let-7e/125a and miR-212/132 clusters impairs osteoclastogenesis

**Figure 2:**
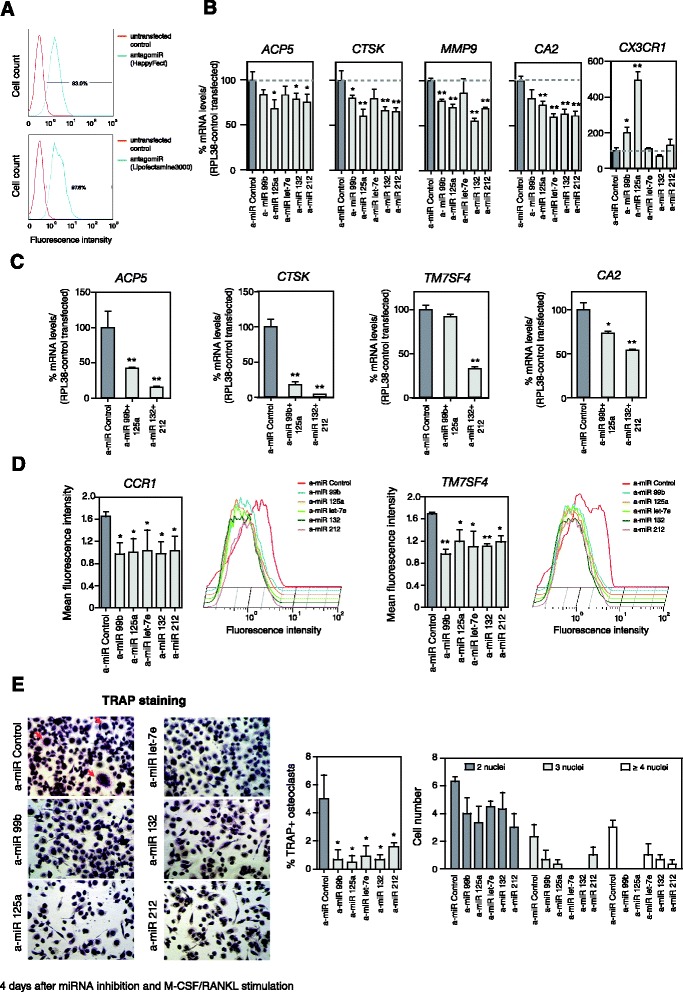
**Influence of miRNAs in modulating monocyte-to-osteoclast differentiation. (A)** Quantification by flow cytometry of the transfection efficiency using a fluorescent control power inhibitor or antagomir. **(B)** Functional effect of miRNA inhibition using power inhibitors (or antagomirs) for the individual miRNAs in the miR-99b/125a/let7e and miR-132/212 clusters on *CA2*, *CTSK*, *MMP9*, *ACP5* and *CX3CR1* expression levels 4 days after M-CSF/RANL stimulation. Quantification was done using qRT-PCR with specific primers for each gene and using the *RPL38* gene for normalization. **(C)** Functional effect of miRNA inhibition using double transfections with power inhibitors for two miRNAs within the miR-99b/125a/let7e and miR-132/212 clusters. Quantification was carried out using qRT-PCR with specific primers for each gene and using the *RPL38* gene for normalization. **(D)** Effect of miRNA inhibition on the levels of surface markers CCR1 and TM7SF4. A bar diagram summarizing the results of the individual inhibition of each miRNA of the two clusters is presented. Also, a plot of the fluorescence-activated cell sorting (FACS) analysis is presented. **(E)** Effect of miRNA inhibition on the ability of cells to differentiate in OCs. Cells were arrested at 4 days after inducing differentiation. OCs were stained with TRAP. Cells with three or more nuclei were counted as OCs. In the images, multinuclear OCs are indicated with a red arrow. On the right, a bar diagram showing the percentage of OCs under each condition (center) and a bar diagram showing the number of cells with two, three or four or more nuclei under each condition (right). Error bars correspond to the standard deviation of three independent measurements; *corresponds to P-value <0.05; **means P-value <0.01.

To investigate the role of the individual miRNAs within the two aforementioned clusters in OC differentiation, we performed loss of function experiments. We transfected primary MOs with specific inhibitors or antagomirs for each of the individual miRNAs contained in the miR-99b/let-7e/125a and miR-212/132 clusters. In these experiments, transfections with miRNA inhibitors were performed simultaneously with RANKL/M-CSF stimulation. We collected samples at 4 days. Then we tested the expression of OC markers to assess the impact of depleting these miRNAs on the differentiation process. Simultaneously, we checked the efficiency of transfection by flow cytometry of cells transfected with a control antagomir fluorescein-conjugate, recording efficiencies of 93% and 97.6% depending on the reagent used for transfection (Figure 2A). qRT-PCR analysis revealed that individual inhibition of each of the miRNAs within the two clusters results in a delay and decrease in the levels of OC markers like *ACP5*, *CA2*, *CTSK* and *MMP9* (Figure 2B) at 4 days after RANKL/M-CSF stimulation. An opposite effect was observed for the MO-specific gene *CX3CR1* with miR-99b and miR-125a (Figure 2B). We also performed double-transfection experiments with two combinations of miRNA inhibitors, containing two miRNAs within each cluster. In these experiments with two miRNA inhibitors we observed higher inhibition of OC markers than in single transfections (Figure 2C), further demonstrating the functional role of these miRNAs in the proper differentiation of OCs.

We then investigated the effects of depleting these miRNAs on the acquisition of two essential OC membrane proteins, CCR1 [27] and TM7SF4/DCSTAMP [28] (Figure 2D). Flow cytometry analysis of these two surface markers revealed that the depletion of any of the individual miRNAs within the two clusters decreases their levels 4 days after RANKL/M-CSF stimulation.

Finally, we tested whether depletion of these miRNAs impacts the ability of MOs to fuse and form OCs following RANKL/M-CSF stimulation. To this end, we performed TRAP staining 4 days after RANKL/M-CSF treatment on cells transfected with power inhibitors for each of the miRNAs, when the first multinucleated OCs start to be apparent. We observed a delay in OC formation in all cases, proving the relevance of these miRNAs for the differentiation and fusion in TRAP+ OCs (Figure 2E). These effects were less obvious at longer differentiation times; however, this is not surprising given the medium/long-term instability of antagomirs transfected into cells. In summary, all these results indicate that the high levels of the miRNAs from these two clusters are necessary for proper differentiation of OCs.

### Upregulated miRNAs target monocyte-specific and immunomodulatory genes that need to be silenced during osteoclastogenesis

**Figure 3:**
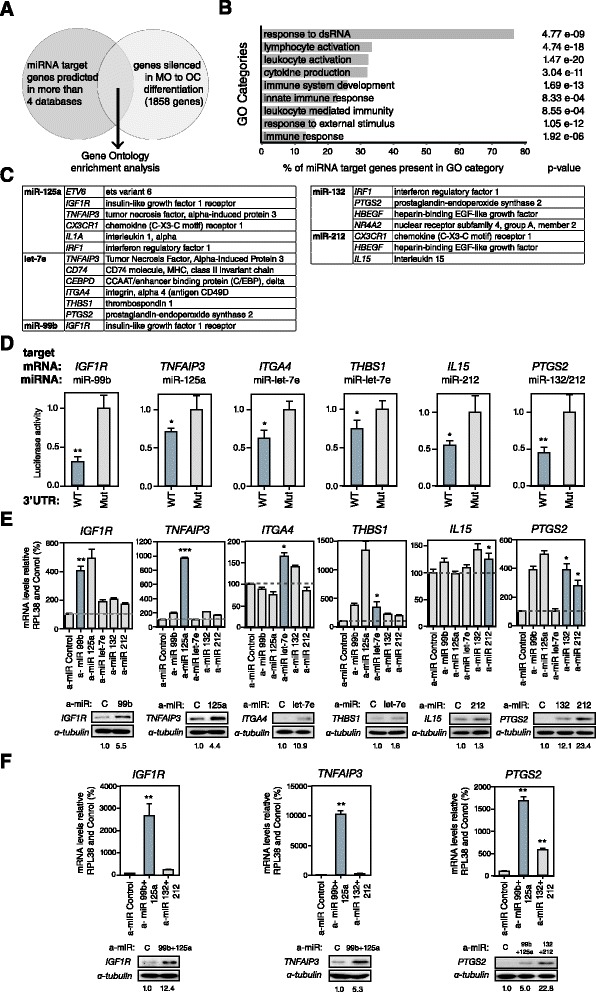
**Analysis of miRNA targets. (A)** Venn diagram summarizing the rationale for selecting putative miRNA targets by combining the lists generated with prediction algorithms with those generated from expression datasets (1,858 genes with a fold change <0.5). **(B)** Gene Ontology (GO) enrichment analysis of putative miRNA targets from the previous analysis. **(C)** Summary of putative targets and their corresponding miRNA matches among the miR-99b/125a/let7e and miR-132/212 clusters. **(D)** Luciferase assays of HeLa cells cotransfected with different luciferase reporter psiCheck2 constructs containing the 3′ UTR of putative targeted transcription factors (wild type (WT) or mutant (Mut) forms). **(E)** Effects on validated targets of the single transfection with miRNA power inhibitors in MOs 4 days after being stimulated with RANKL/M-CSF, as assessed by qRT-PCR and western blotting. Expression data are relative to the levels obtained for the samples transfected with control power inhibitor or antagomir (a-miR) and are normalized to the *RPL38* gene. Protein data have been normalized against α-tubulin, using the sample transfected with the control power inhibitor as a reference. At the bottom, quantification of the levels of protein relative to the control for each antagomir. **(F)** Effects on validated targets of the double transfection with miRNA power inhibitors in MOs 4 days after being stimulated with RANKL/M-CSF, as assessed by qRT-PCR and western blotting. Data analyzed as above. Error bars correspond to standard deviation of three independent experiments; *corresponds to P-value <0.05; **means P-value <0.01; ***means P-value < 0.001.

Our results demonstrated that the miRNAs within the most strongly activated miRNA clusters have a functional effect on OC differentiation when inhibited in MOs, as reflected by the decrease and delay in the upregulation of OC markers, including OC-specific surface proteins, as well as in the ability to form multinuclear cells. To identify the targets of these miRNAs, we retrieved a list of putative targets using miRWalk [29], which contains prediction databases like TargetScan [30], miRDB [31] and others, as well as information about validated targets. We then linked the list of potential targets with previously reported high-throughput data on expression changes during OC differentiation [32], assuming an inverse relationship between the levels of a given miRNA and the expression levels of its targets. For this analysis, we imposed the criteria that the targets should be predicted by at least four databases (Figure 3A) and that downregulation was defined as a minimum 0.5-fold change. Applying these conditions we identified a number of putative downregulated targets for each overexpressed miRNA (Additional file 2). We then used the Database for Annotation, Visualization and Integrated Discovery (DAVID) to identify functional categories. This tool revealed a highly significant enrichment of categories related to the immune system (Figure 3B), including immune system development (*P*-value 1.69 E-11) and cytokine production (*P*-value 3.04 E-11). We identified a number of critical factors from among these (Figure 3C). For instance, miR-99b was found to target the 3′ UTRs of *IGF1R*, miR-125a targeted *ETV6*, *TNFAIP3* and *CX3CR1*, and let7e targeted *TNFAIP3* and *ITGA4*. In the case of the miRNAs in the miR-212/132 cluster, miR-212 was found to target *CX3CR1* and *HBEGF*, and miR-132 targeted *IRF1* and *NR4A2*. Some of these genes are also silenced by other mechanisms during OC differentiation. Two examples are the MO-specific gene *CX3CR1* and the anti-inflammatory gene *TNFAIP3*, which are rapidly silenced after MCSF/RANKL stimulation, and their promoters are hypermethylated [33].

To validate the putative targets, we performed luciferase reporter assays using a vector containing the renilla luciferase coding region plus the wild type or a mutant form (Mut) of the putative 3′ UTR target sites of each potentially targeted gene. We carried out these assays in HeLa cells, in which we had previously estimated the expression of high levels of miRNAs of the miR-99b/let-7e/125a and miR-212/132 clusters. These assays confirmed that miR-99b targets *IGF1R*, miR-125a targets *TNFAIP3*, and let-7e targets *ITGA4* and *THBS1*. On the other hand, miR-132 targets *PTGS2*, and miR-212 also targets *PTGS2* and *IL15* (Figure 3D).

To obtain further evidence of the *in vivo* effect of the miRNAs on their putative targets, we performed qRT-PCR and western blotting in MOs transfected with each of the miRNA inhibitors. In the case of the miR-99b/let-7e/125a cluster, inhibition of miR-99, miR-125a and let-7e resulted in the specific upregulation of *IGF1R*, *TNFAIP3* and *IGF1R*, and of *ITGA4* and *THBS1*, respectively. With respect to the miR-212/132 cluster, inhibition of miR-132 and miR-212 gave rise to the upregulation of *PTGS2* and the inhibition of miR-212 resulted in the upregulation of *IL15* (Figure 3E). We also observed crossover effects between some miRNAs and targets. For instance, inhibition of miR-99b and miR-125a also affected *PTGS2*, which was not validated in luciferase assays but also contains putative recognition sites at its 3′ UTR for miR-99b and miR-125. We observed increased mRNA and protein levels for some of these targets in double transfection experiments with antagomirs (Figure 3F). Together with the luciferase assays, all these results confirmed the essential role of the miRNAs in the downregulation of these genes during OC differentiation.

### Changes in the miRNA cluster expression levels in related inflammatory-driven monocyte differentiation processes

**Figure 4:**
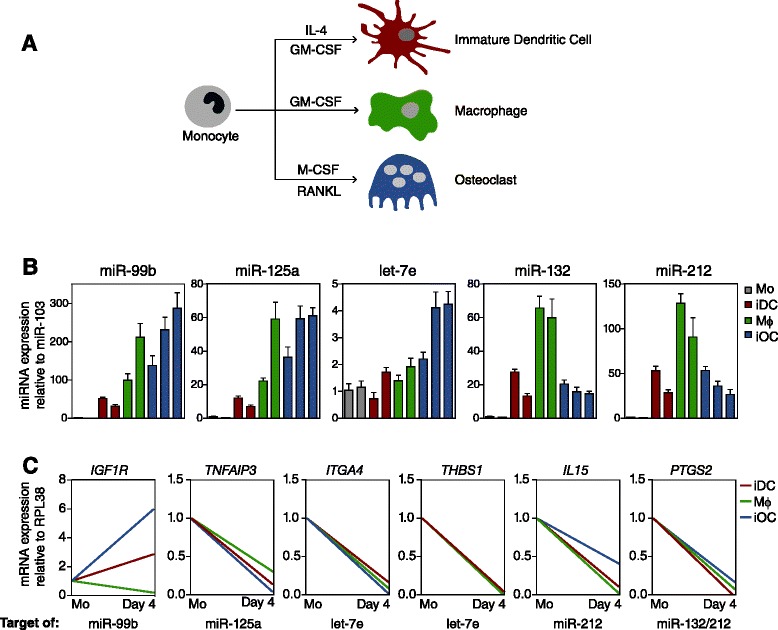
**Comparison of changes in the expression levels of the miRNAs within the miR-99b/125a/let7e and miR-132/212 clusters during osteoclast differentiation, and changes during monocyte-to-macrophage and monocyte-to-dendritic cell differentiation. (A)** Diagram depicting the three differentiation models used in this experiment. GM-CSF, granulocyte-macrophage colony-stimulating factor. **(B)** Relative expression levels of the miRNAs in matching samples of MOs (grey), immature dendritic cells (iDC, red), macrophages (green,) and monocytes stimulated with RANKL/M-CSF after 1, 2 and 4 days (immature OCs (iOC) (blue)). qRT-PCR data were normalized with respect to miR-103. **(C)** Relative expression levels of miRNA targets in the same set of samples. qRT-PCR data were normalized with respect to the *RPL38* gene. Error bars correspond to standard deviation of three independent measurements.

We also investigated whether the observed miRNA expression changes occurring in OC differentiation constitute a more general regulatory mechanism also operating in another two related differentiation processes involving MOs, specifically MO-to-dendritic cell differentiation and MO-to-macrophage differentiation. These two processes are triggered following stimulation with granulocyte-macrophage CSF/IL4 or granulocyte-macrophage CSF alone (Figure 4A). Analysis of the expression changes of all miRNAs within the miR-99b/let-7e/125a and miR-212/132 clusters showed these are common to all three processes (Figure 4B). Specifically, we observed that all these miRNAs increased more markedly in macrophages than in dendritic cells, suggesting a bias towards the ability to generate a strong NF-κB-mediated response, such as TLR4-initiated signals occurring in inflammatory macrophages. Given the negative relationship between these miRNAs and the regulation of their targets, it is feasible that they have a key role in the extinction of mRNAs that are characteristic of a less inflammatory prone state. This prompted us to investigate the relationship between the increase in these miRNAs and the expression levels of their validated targets in MO-to-dendritic cell and MO-to-macrophage differentiation. We also noted a decrease in the mRNA levels of *TNFAIP3*, *ITGA4*, *THBS*, *IL5*, and *PTGS2* during the three differentiation processes (Figure 4C). The only exception was *IGF1R*, which appeared to increase over time in OC and dendritic cell differentiation, consistent with the findings of others [34]. This could perhaps be due to the predominant effect of other regulatory mechanisms, probably at the transcription level (Figure 4C). In this case, the upregulation of miR-99b, which targets *IGF1R*, could be related more to fine-tuning regulation in order to achieve the proper levels of this protein instead of blocking the expression of it.

### A direct role for NF-κB in the upregulation of miRNAs?

**Figure 5:**
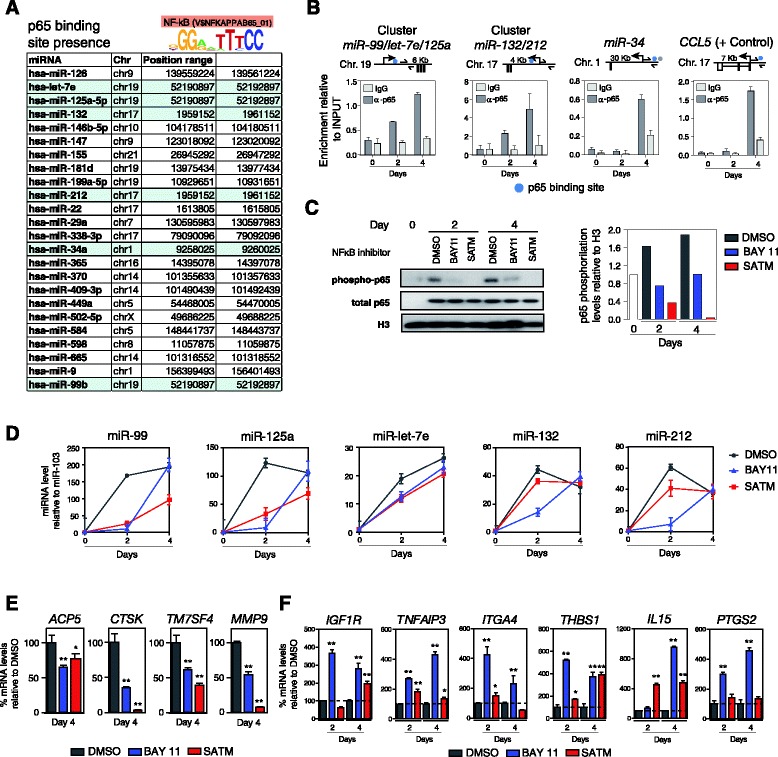
**NF-κB dependence of miRNA expression changes. (A)** Analysis of the presence of NF-κB subunit binding motifs (from TRANSFAC database) in a 1,000-bp window centered around the estimated TSS of the miRNAs. **(B)** ChIP assays for selected miRNAs showing the binding of NF-κB p65 near the TSS 2 and 4 days after RANKL/M-CSF stimulation of MOs. Each graph contains the relative enrichment of samples immunoprecipitated with the anti-p65 antibody and an IgG as a control. MO samples were tested at 0, 2 and 4 days after M-CSF/RANKL stimulation. On top of each graph the sequence analyzed is indicated. p65 putative binding sites are indicated with a blue dot. Primers used for amplification are indicated with arrows around p65 binding sites. **(C)** Effects of the two NF-κB inhibitors (10 μM BAY 11-7082 (BAY11) and 100 μM sodium aurothiomalate (SATM) on the phosphorylation levels of p65 as determined by western blotting. p65 and H3 total levels are used as controls. **(D)** Effects of two NF-κB inhibitors (BAY11 and SATM) on the levels of miRNAs within the miR-99b/125a/let7e and miR-132/212 clusters measured by a time-course analysis of MOs stimulated with RANKL/M-CSF. **(E)** Effects of treatment with BAY11 and SATM on markers of OC differentiation (*ACP5*, *CTSK*, *TM7SF4*, *MMP9*) as estimated by qRT-PCR. Data relative to DMSO-treated samples and normalized with *RPL38* expression levels. **(F)** Effects of treatment with BAY 11 and SATM on miRNA targets (*IGF1R*, *TNFAIP3*, *ITGA4*, *THBS*, *IL15* and *PTGS2*) as estimated by qRT-PCR. Data are relative to DMSO-treated samples and are normalized with respect to *RPL38* expression levels. Error bars correspond to the standard deviation of three independent measurements; *corresponds to *P*-value <0.05; **means *P*-value <0.01.

Our results supported the notion that miRNAs play a role in the efficiency of OC differentiation and enabled us to identify two upregulated miRNA clusters whose participation in downregulating genes is key to this process. As explained above, MO-to-OC differentiation is induced by RANKL, which ultimately stimulates NF-κB, a transcription factor once it has been translocated into the nucleus. NF-κB acts in concert with PU.1, Jun and the OC-specific transcription factor NFATc1. NF-κB helps regulate many OC-specific genes. The transcription factor NF-κB is also likely to participate in shutting down MO-specific genes through the activation of miRNAs. To explore the potential involvement of NF-κB in the changes in miRNAs during MO-to-OC differentiation, we first investigated the enrichment of the consensus binding site for the p65 NF-κB subunit in a window of 500 bp upstream and downstream of the TSS of the miRNAs (determined from the miRStart database) [35]. This analysis showed that the p65 NF-κB consensus binding site is present in the majority of miRNA TSSs, including the miRNAs within the miR-99b/let-7e/125a and miR-212/132 clusters (Figure 5A). We then investigated the presence of p65 NF-κB around the TSSs of these two miRNA clusters by performing chromatin immunoprecipitation (ChIP) assays with anti-p65 antibodies in MOs before and 48 h and 96 h after induction with RANKL/M-CSF. We also used primers near the TSS of *CCL5* as a positive control. We noted specific enrichment of p65 at 48 h and 96 h after RANKL/M-CSF stimulation in the two upregulated miRNA clusters (Figure 5B), demonstrating a direct association of NF-κB p65 with the encoding sequence of the upregulated miRNAs. We also found that p65 bound near the TSSs of other miRNAs, such as miR-34a (Figure 5B), suggesting that this may be a general mechanism of miRNA upregulation in OC differentiation.

To investigate the involvement of the NF-κB pathway in the activation of these miRNAs in greater depth, we treated MOs with two NF-κB inhibitors, Bay 11-7082 and sodium aurothiomalate (SATM), and investigated the effects on the expression of the aforementioned miRNAs following induction by RANKL/M-CSF. SATM inhibits the activity of IκB kinase by modifying cysteine residues within its catalytic domain. Bay 11-7082 selectively and irreversibly inhibits the tumor necrosis factor-α-inducible phosphorylation of IκBα, resulting in reduced expression of NF-κB and adhesion molecules. Both inhibitors eventually reduce the levels of phosphorylated Ser536 of p65, which correspond to its active form. To test the toxicity of these two inhibitors, we first performed MTT assays over a wide range of concentrations with primary MOs (not shown), and selected 10 μM for Bay 11-7082 and 100 μM for SATM. Consistent with a relevant role of the NF-κB pathway in the activation of these miRNAs, we observed that phosphorylation of p65 increases following RANKL/M-CSF stimulation of MOs (Figure 5C). Under our conditions we observed potent inhibition of p65, as reflected by the reduced levels of phospho-Ser536 p65, especially at 2 days, whereas at 4 days the inhibitory effects of Bay 11-7082 were significantly reduced, perhaps due to its instability in the culture medium (Figure 5C). We then investigated the effects of these two inhibitors on miRNA expression. Both inhibitors decreased expression of upregulated miRNAs (Figure 5D), reinforcing the notion of the direct role of NF-κB in mediating their upregulation. Consistent with the results obtained with the western blotting (Figure 5C), the reduction in miRNA upregulation was more obvious only at 2 days, whereas at 4 days the miRNAs of cells treated with Bay 11-7082 had reached the levels of cells treated with the vehicle. As mentioned above, this is perhaps due to the stability of the inhibitors in the culture medium, which are added only at the beginning. It could also be due to the contribution of additional regulatory mechanisms that could be compensating for the inhibition of the NF-κB pathway. We also checked the effects on both classical OC markers and miRNA-validated targets. The two inhibitors reduced the levels of OC markers, as determined by qRT-PCR after 4 days (Figure 5E). Conversely, both SATM and Bay 11-7082 had an overall positive effect on miRNA targets, providing evidence that NF-κB helps repress these targets through miRNAs (Figure 5F). We observed different effects in terms of which of the two drugs was more effective or whether the effect was more evident at 2 or 4 days, but we cannot discard the occurrence of pleiotropic effects, or interference with other regulatory mechanisms.

**Figure 6:**
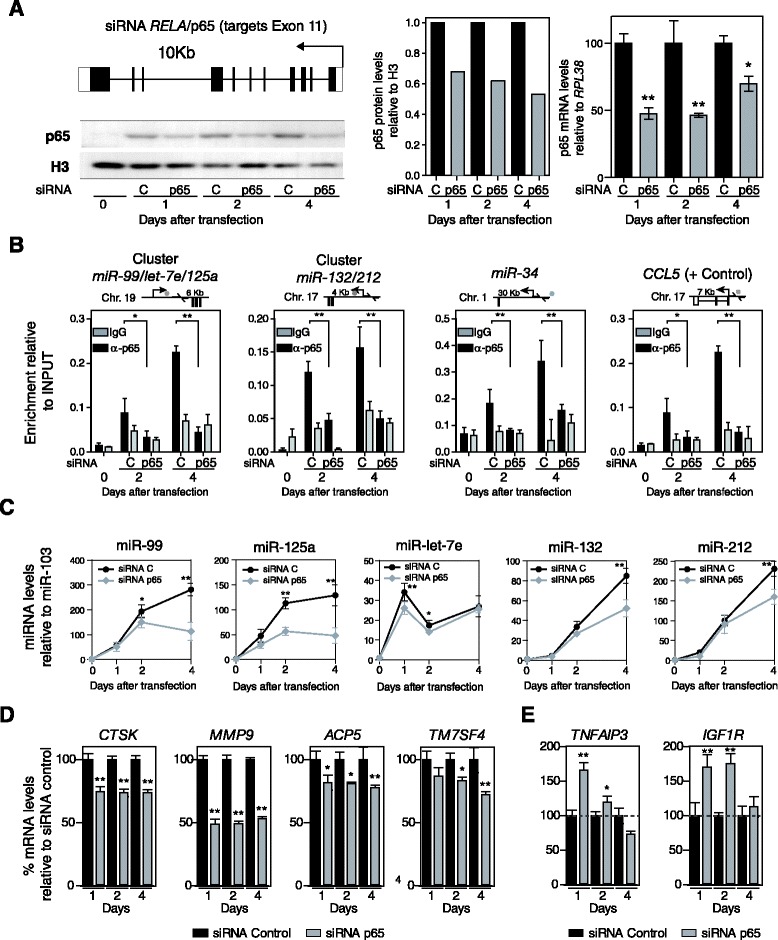
**NF-κB p65 has a direct role in changes in miRNA expression levels. (A)** Diagram depicting the region of the p65 gene in exon 11 targeted by the siRNA used in this study. Effects of siRNA experiments on p65 levels in MOs stimulated with RANKL/M-CSF after 1, 2, and 4 days, as analyzed by western blotting (bottom and central panels, normalized with respect to histone H3 levels) and qRT-PCR (left panel, relative to RPL38 expression levels). **(B)** Effect of p65 depletion on its recruitment near the TSS of the coding sequence of the miRNAs, as demonstrated by ChIP assays. The scheme on top of each graph depicts the region analyzed, indicating the p65 binding site (dot) and the primers used (arrows around the p65 binding site). **(C)** Effects of p65 siRNA experiments on miR-99b, miR-125a and miR-let7e after 1, 2, and 4 days. Data relative to miR-103 levels. **(D)** Effects of p65 downregulation on expression of genes upregulated during osteoclastogenesis (*CTSK*, *MMP9*, *ACP5*, *TM7SF4*). **(E)** Effects of p65 downregulation on the levels of the miRNA targets *TNFAIP3* and *IGF1R*. Expression data compared with MO samples treated with control siRNA and values relative to *RPL38* expression levels. Error bars correspond to the standard deviation of three independent measurements; *corresponds to *P*-value <0.05; **means *P*-value <0.01.

Therefore, as an unequivocal test of a potential causal relationship between NF-κB and miRNA expression changes in MO-to-OC differentiation, we investigated the consequences of ablating p65 expression in MOs. To this end, we downregulated p65 levels in MOs using transient transfection experiments with a small interfering RNA (siRNA) that targets exon 11 of p65 (Figure 6A). In parallel, we used a control siRNA. Following transfection we stimulated differentiation with RANKL/M-CSF. Under these conditions, we used qRT-PCR and western blotting to check the effects on p65 levels 1, 2 and 4 days after RANKL/M-CSF stimulation of MOs. By this means, we were able to confirm that the level of p65 downregulation was close to 50% (Figure 6A). siRNA-mediated downregulation of p65 resulted in decreased binding of the miRNAs to TSSs (Figure 6B). We also examined the expression levels of these miRNAs following p65 depletion and found that the RANKL/M-CSF-stimulated upregulation of the miRNAs within the miR-99b/let-7e/125a cluster was partially impaired following p65 depletion (Figure 6C). We also analyzed two miRNAs that are not direct p65 targets and observed no effect on their levels following p65 depletion (not shown).

We investigated the effects of depleting p65 on the expression of OC markers (*ACP5*, *CTSK* and *TM7SF4*) as well as on miRNA targets. Whereas depletion of p65 led to a decrease in the upregulation of OC markers (Figure 6D), it resulted in an increase in miRNA targets (Figure 6E), thereby confirming the direct role of p65 in regulating this process.

Taken together, our findings are the first demonstration that NF-κB is directly associated with and activates miRNAs that are essential for the regulation of critical targets whose downregulation is essential for proper OC differentiation.

## Discussion

Our results provide novel insights into the role and mechanisms of the fine-tuned control of expression and its relation with inflammatory pathways in MO-to-OC differentiation. Firstly, we identified a set of miRNAs that are required for OC differentiation. Most importantly, these miRNAs target and repress OC-inappropriate genes, including several MO-specific and immunomodulatory genes. Secondly, our results reinforce the key role of the NF-κB transcription factor as a direct regulator of miRNA upregulation, specifically focusing on the miR-99b/let-7e/125a and miR-212/132 clusters.

Screening miRNA expression changes at two points during differentiation revealed different groups of miRNAs based on their expression profiles over time. Overall, our data show prevalent upregulation of miRNAs in OC differentiation. Twenty-three miRNAs displayed fast upregulation followed by sustained expression levels, 20 miRNAs had a rapid increase followed by downregulation over a longer period following induction until day 21, and 26 miRNAs were upregulated only at later stages. In contrast, there were significantly fewer miRNAs whose expression levels decreased over time. The predominance of upregulated miRNAs may suggest that their primary role is to repress or ensure the maintenance of low levels of OC-inappropriate genes that could also be repressed through other mechanisms. Previous data from our group have already shown this type of behavior in B cell-to-macrophage transdifferentiation [36]. Our analysis of the functional effects of the depletion of the miRNAs within the miR-99b/let-7e/125a and miR-212/132 clusters, as well the analysis of their targets, shows that these molecules have a direct role in repressing MO-specific and immunomodulatory genes like *TNFAIP3*, *IGF1R* and *IL15*. In addition, loss of function experiments using specific inhibitors for the above miRNAs influences the efficiency of osteoclastogenesis, as determined by analyzing expression changes of standard markers of OC differentiation at the RNA and protein levels, the effects on validated targets of these miRNAs and the ability of cells to fuse to yield multinucleated OCs.

Our results suggest that fine-tuning modulation through miRNA-mediated repression drives the monocytic steady state program into an NF-κB-driven proinflammatory differentiation program. This idea is reinforced by the observation that the upregulation of these miRNAs also occurs in related inflammatory-related monocytic differentiation processes, including MO-to-dendritic cell differentiation and MO-to-macrophage differentiation. MOs are heterogeneous circulating progenitors that can either patrol the resting endothelium or migrate into tissues in response to inflammatory signals. Regulation of switching between these different states requires the ability to respond rapidly to changes that may include silencing of undesired response pathways, and the commitment to ensure proper outcomes. miRNAs may contribute to this process as a flexible regulatory mechanism, as it has been described for miR146a and Relb pathway Ly6Chigh inflammatory MO responses [37]. Our analysis on the functional effects of depletion of the miRNAs within the miR-99b/let-7e/125a cluster reveals a possible common pathway of commitment into cells with strong NF-κB-dependent responses, suggesting the targeting of the anti-inflammatory molecule TNFAIP3 (A20) by these microRNAs, which are upregulated in the conversion into OCs, dendritics cells and macrophages. In addition, depletion of the miR-212/132 cluster, as well as the analysis of their targets, shows that these elements have a direct role in repressing genes like *IRF1* or *IL15*, which could also shape inflammation.

Ly6Chigh MO conversion to Ly6Clo anti-inflammatory macrophages with a restorative phenotype in murine hepatic fibrosis requires the upregulation of genes encoding molecules of the anti-inflammatory macrophage program, like *CX3CR1*, or with anti-fibrotic effects, like *CD74* [38]. Interestingly, both genes are targeted by the miR-212/132 cluster in our MO-based differentiation models that converge on the set-up of inflammatory or NF-κB programs in different cell types. In addition, an immunosuppresive role has also been assigned to the IGF1R-IGF1 axis, and cord blood mononuclear cells as well as peripheral blood mononuclear cells (PBMCs) treated with IGF1 show a decrease NF-κB binding activity [39]. Our results show that *IGF1R* is targeted by miR99b and miR125a also suggesting a coordinated shutdown of signal transduction that block NF-κB pathways.

The second major conclusion of our study is the role of NF-κB in directly upregulating the miR-212/132 and miR-99b/let-7e/125a clusters, and perhaps other miRNAs. Multiple genes implicated in inflammation, including proinflammatory cytokines and their receptors, are under the transcriptional control of the transcription factor NF-κB [40]. A few reports have recently proved that NF-κB has a direct role in regulating miRNA expression [41,42]. To the best of our knowledge, however, this is the first report demonstrating a direct role for NF-κB in miRNA control in OC differentiation. NF-κB is a major target of RANKL, which is used together with M-CSF to stimulate differentiation of MOs into OCs. However, only a few direct NF-κB targets have so far been described. For instance, it has been shown that NF-κB cooperates with NFATc2 to induce expression of NFATc1, with NFkB p50 and p65 being recruited to the NFATc1 promoter within 1 hour of treatment of OCPs with RANKL, resulting in transient autoamplification of NFATc1 expression, which is crucial for OC formation [14]. To date, the participation of NF-κB in this context had been restricted to an activator of genes that are necessary for OC differentiation. Our findings reveal a novel role for NF-κB in activating miRNAs that repress the expression of OC-inappropriate genes that are not required for differentiation. This perhaps includes not only MO-to-OC differentiation, but also other related MO-related differentiation processes where NF-κB plays a key role. Several papers have come out showing regulatory programs of Ly6Chigh inflammatory MOs/macrophages versus Ly6Clo resting cells [37,38,43]. Nonetheless, unraveling the mechanisms that delineate NF-κB versus other programs in human MOs has been more difficult and this issue is directly addressed by the present work.

The results of our study constitute the first clear evidence that NF-κB directly regulates miRNAs, showing together with our findings on the miRNA targets and the impact on OC differentiation that this is a novel mechanism of gene repression of OC-inappropriate genes in this differentiation process. In addition, our conclusions open up possibilities for exploiting novel pathways for therapeutic intervention.

## Conclusions

Our study on miRNA expression changes during MO-to-OC differentiation reveals the occurrence of rapid upregulation of two miRNA clusters. We have demonstrated that miRNAs within these two clusters are necessary for MOs to differentiate into OCs. These miRNAs are key to repressing OC-inappropriate genes, including certain anti-inflammatory genes, and are needed for proper OC differentiation. We demonstrate that these changes and their functional effects also occur in other MO differentiation processes, indicating that these miRNAs are needed for the downregulation of OC-inappropriate genes in MOs. Most importantly, we demonstrate for the first time that NF-κB directly regulates these miRNAs and is thus directly implicated in the inhibition of the less differentiated monocytic expression program.

## Materials and methods

### Differentiation of osteoclasts from peripheral blood mononuclear cells

Human blood samples came from anonymous blood donors through the Catalan Blood and Tissue Bank in Barcelona as thrombocyte concentrates (buffy coats). The anonymous blood donors received oral and written information about the possibility that their blood would be used for research purposes, and any questions that arose were then answered. Before giving their first blood sample the donors signed a consent form at the Banc de Teixits, which adheres to the principles set out in the WMA Declaration of Helsinki. The blood was carefully layered on a Ficoll-Paque gradient (Amersham, Buckinghamshire, UK) and centrifuged at 2,000 rpm for 30 minutes without braking. After centrifugation, PBMCs at the interface between the plasma and the Ficoll-Paque gradient were collected and washed twice with ice-cold phosphate-buffered saline, followed by centrifugation at 2,000 rpm for 5 minutes. Pure CD14+ cells were isolated from PBMCs using positive selection with MACS magnetic CD14 antibody (Miltenyi Biotec, Bergisch Gladbach, Germany). Cells were then resuspended in α-minimal essential medium (α-MEM GlutaMAX Supplement, no nucleosides; Invitrogen, Carlsbad, CA, USA) containing 10% fetal bovine serum, 100 units/ml penicillin, 100 μg/ml streptomycin and antimycotic, supplemented with 25 ng/ml human M-CSF and 50 ng/ml soluble hRANKL (PeproTech EC, London, UK). Depending on the amount needed, cells were seeded at a density of 3 × 10^5^ cells/well in 96-well plates, 5 × 10^6^ cells/well in 6-well plates or 40 × 10^6^ cells in 10-mm plates and cultured for 21 days (unless otherwise noted); media and cytokines were changed twice a week. The presence of OCs was checked by TRAP staining using the Leukocyte Acid Phosphatase Assay Kit (Sigma-Aldrich, St. Louis, Missouri, USA) according to the manufacturer’s instructions. Phalloidin/DAPI staining enabled us to confirm that the populations were highly enriched in multinuclear cells, some of which contained more than 40 nuclei. We used several methods to determine that on day 21 almost 85% of the nuclei detected were ‘osteoclastic nuclei’ (in polykaryons; nuclei, rather than cells, were quantified). OCs (TRAP-positive cells with three or more nuclei) were also analyzed at the mRNA level: upregulation of key OC markers (*CA2*, *CTSK*, *MMP9*, *ACP5/TRAP* and *TM7SF4/DCSTAMP*) and downregulation of the MO marker *CX3CR1* were confirmed.

### Visualization of osteoclasts with phalloidin and DAPI staining

Pure isolated CD14+ cells were seeded and cultured in glass Lab-Tek Chamber Slides (Thermo Fisher Scientific, Waltham, MA, USA) for 21 days in the presence of human M-CSF and human RANKL. OCs were then washed twice with phosphate-buffered saline and fixed (3.7% paraformaldehyde, 15 minutes). Cells were permeabilized with 0.1% (V/V) Triton X-100 for 5 minutes and stained for F-actin with 5 U/ml Alexa Fluor® 647-Phalloidin (Invitrogen). Cells were then mounted in Mowiol-DAPI mounting medium. Cultures were visualized by confocal laser scanning microscopy (Leica TCP SP2 AOBS confocal microscope).

### Flow cytometry

Cells were stained with fluorochrome-conjugated antibodies against CCR1 (R&D Systems, reference FAB145A-100) and TM7SF4 (R&D Systems, reference FAB7824-A) (Both antibodies are from R&D Systems, Minneapolis, MN, USA) 0 and 4 days after RANKL/M-CSF stimulation. CCR1 and TM7SF4 expression were monitored on a Gallios Flow Cytometer (Beckman Coulter, Pasadena, California, USA) and analyzed by FlowJo software (Tree Star, Inc., Ashland, Oregon, USA). All experiments were performed in triplicate and bar graphs correspond to independent biological samples.

### MicroRNA expression screening and target prediction

Total RNA was extracted with TriPure (Roche, Basel, Switzerland) following the manufacturer’s instructions. Ready-to-use miRNA PCR Human Panel I V2.R from Exiqon (reference 203608) was used according to the instruction manual (Exiqon, Vedbeak, Denmark). Total RNA (30 ng) was used for each RT-PCR reaction. Paired samples of MOs at 0 (MO), 2 (OC 48 h) and 21 (OC) days after M-CSF and RANKL stimulation were obtained from three female healthy donors (aged 25 to 28 years), and were analyzed with a Roche LightCycler® 480 real-time PCR system. Results were converted to relative values using the inter-plate calibrators included in the panels (log_2_ ratios). Average expression values of MO, OC 48 h and OC were normalized with respect to the reference gene miR-103. A *t*-test was then performed and differentially expressed miRNAs (fold change >2 or <0.5), with a significant *P*-value (*P* < 0.05) in at least one of the comparisons, were selected and represented on a heatmap. The raw expression data are listed in full in Additional file 1. The array expression data were validated in the samples used (validation set), and in a larger cohort of samples obtained from independent donors (replication set) using Exiqon microRNA LNA™ PCR primer sets (hsa-miR-99b-5p, reference 204367; hsa-miR-125a-5p, reference 204339; hsa-miR-132-3p, reference 204129; hsa-miR-212-3p, reference 204170; hsa-miR-103a-3p, reference 204063).

To predict the potential targets of the deregulated miRNAs, we used the algorithms from several databases: TargetScan, PicTar, PITA, miRBase, microRNA.org, miRDB/MirTarget2, TarBase, miRecords and StarBase/CLIPseq. Only targets predicted by at least four of these databases were retained for further analysis.

### Bioinformatics analysis of expression data

To compare the expression data with the methylation data, we used CD14+ and OC expression data from the ArrayExpress database [44] (accession EMEXP-2019) from a previous publication [32]. Affymetrix GeneChip Human Genome U133 Plus 2.0 expression data were processed using the limma and affy packages from Bioconductor. The pre-processing stage was divided into three main steps: background correction, normalization and reporter summarization. We chose the expresso function of the affy package for preprocessing. Thus, the robust multi-array average (RMA) method was used for background correction. Quantile normalization was then done. We also introduced a specific step for PM (perfect matchprobes) adjustment, using the PM-only model-based expression index (option ‘pmonly’). Finally, for the summarization step, the median polish method was used. Next, variance filtering by IQR (interquartile range) was carried out, taking 0.50 as the threshold value. After preprocessing, data were analyzed using the empirical Bayes moderated *t*-test available in the limma statistics package. Expression data were validated by qRT-PCR.

### Transfection of primary human monocytes with miRNA inhibitors and p65 NF-κB siRNA

To perform the miRNA inhibitor experiments, we used unlabeled miRCURY LNA™ microRNA Power inhibitors to inhibit miR-99b (reference 4101513), miR-let-7e (reference 4103550), miR-125a (reference 4103094), miR-132 (reference 4103093), miR-212 (reference 4104787) or a control (Negative Control A, reference 199006) Exiqon, Vedbaek, Denmark. Power inhibitors (5 or 10 nM) were transfected into CD14+ MOs using HappyFect Transfection Reagent (Tecan, Weymouth, UK) or Lipofectamine ®3000 (Life Technologies). Cells were simultaneously incubated in the presence of RANKL/M-CSF in the conditions previously described. The efficiency of transfection was quantified by flow cytometry using the 5′-fluorescein-labeled Negative Control A. For samples collected at 4 days or after, we added a fresh aliquot of miRNA inhibitors after 48 h. To silence p65, we used Silencer® Select Pre-Designed siRNA (Life Technologies) against human RELA (p65), targeting exon 11 (reference s11916) in parallel with a Silencer® Select negative control in purified CD14+ MOs in the presence of M-CSF, followed by stimulation with RANKL (and M-CSF) 24 h after siRNA transfection. We used Lipofectamine RNAiMAX Transfection Reagent (Invitrogen) for efficient siRNA transfection. mRNA and protein levels were examined by qRT-PCR and western blotting 1, 2, and 4 days after siRNA transfection. These experiments were performed with at least three biological replicates.

### Luciferase assays

The putative miRNA binding sites in the 3′ UTRs of *IGF1R*, *TNFAIP3*, *ITGA4*, *THBS1*, *IL15*, and *PTGS2* were amplified by PCR from genomic DNA derived from CD14+ cells. The PCR products were cloned into pGEM®-T Easy Vector (Promega, Madison, Wisconsin, USA) and four to seven point mutations were introduced into each target site by site-directed mutagenesis. Each of the fragments containing the 3′ UTR of putative miRNA binding sites was cloned into psiCHECK-2 vector (Promega). 293 T cells were cultured for 24 h and then co-transfected using lipofectamine RNAimax with 10 ng of psiCHECK-2 vector containing wild-type or mutant 3′ UTR plus 50 nM of miRNA power inhibitors per well. The luciferase analysis was performed 48 h later using the Dual-Luciferase Reporter Assay (Promega). Primers to clone the 3′ UTR of putative miRNA binding sites are listed in Additional file 3.

### Chromatin immunoprecipitation assays

For ChIP assays, CD14+ cells 0, 2 and 4 days after treatment with M-CSF and RANKL were crosslinked with 1% formaldehyde and subjected to immunoprecipitation after sonication. ChIP experiments were performed as described elsewhere [33]. Analysis involved real-time qPCR. Data are represented as the ratio of bound fraction to input for each specific factor. We used a mouse monoclonal antibody against the carboxyl terminus of human NF-κB p65 (sc-372, Santa Cruz Biotechnology, Dallas, Texas, USA). Primer sequences are shown in Additional file 3. Experiments included three biological replicates.

### Quantitative RT-PCR and western blotting

RNA was isolated by TRIzol extraction (Invitrogen) and reverse-transcribed using SuperScriptTM II Reverse Transcriptase (Invitrogen). Primers for conventional and qRT-PCR were designed using Primer3 v.0.4.0 (Table S1 in Additional file 1). qRT-PCR was performed in triplicate using LightCycler 480 SYBR Green Mix (Roche). PCR reactions were run and analyzed using the LightCycler 480 II System (Roche). Expression values were normalized against the expression of the endogenous gene controls *RPL38*, *HPRT1* and *GAPDH*. Primers are listed in Additional file 3.

For western blots, protein lysates were generated and western blotting performed using standard procedures using antibodies against phospho-Ser536 p65 (Cell Signaling, 3033 Danvers, Massachusetts, USA), p65 (Santa Cruz Biotechnologies, sc-372), IGF1R (Abcam, ab32823, Cambridge, UK), PTGS2 (Abcam, ab15191), TNFAIP3 (Abcam, ab92324), IL15 (Abcam, ab7213), ITGA4 (Abcam, ab81280), THBS1 (Thermo Scientific, MA5-13398), α-tubulin (Sigma, 1142) and total histone 3 (Abcam, ab1791).

### Graphs and heatmaps

All graphs were created using Prism5 Graphpad (GraphPad Software, San Diego, California, USA). Heatmaps were generated from expression or methylation data using the Genesis program (Graz University of Technology).

### Data access

Raw data for microRNA expression profiling as obtained following qRT-PCR amplification of Ready-to-use microRNA PCR Human Panel I V2.R from Exiqon (reference 203608) is available in Additional file 1. It is also available in NCBI’s Gene Expression Omnibus through GEO Series accession number GSE63773.

## Additional files

Additional file 1:
**Raw data of miRNA expression profiling following quantitative RT-PCR (qRT-PCR) amplification of Ready-to-use microRNA PCR Human Panel I V2.R from Exiqon (reference 203608) in a Roche LC480 II corresponding to pure CD14+ cells (MOs), and these cells at 48 h and 21 d after RANKL/M-CSF stimulation from three anonymous individuals (D1, D2 and D3).** Data are as follows: RAW crossing point (Cp) values as produced by the qPCR system (columns B, D, F, N, P, R, Z, AB, AD), RAW concentration (Conc) as produced by the qRT-PCR system (columns C, E, G, O, Q, S, AA, AC, AE), concentration values relative to miR103 (housekeeping) levels (columns H, I, J, T, U, V, AF, AG, AH). The average of the three samples for each cell type (MOs, OCs 48 h, OCs 21 d) (column K, W, AI), standard deviation (column L, X, AJ). Additional file 2:
**(A)** **Heatmaps corresponding to putative targets for all miRNAs within the miR-99b/125a/let7e and miR-132/212 clusters using miRWalk.** For a given prediction database (DIANAmT, miRanda, miRDB, miRWalk, PICTAR4, PICTAR5, PITA, RNA22, TargetScan) red corresponds to a positive match and blue indicates that it is not predicted. Only those putative targets predicted with at least four algorithms were used. **(B)** Overlap between the analysis with miRWAlk and expression data [32], using those genes that are downregulated in OC differentiation at least 0.5-fold. **(C)** Schematic representations of the pairing between different miRNAs and the 3′ UTR of different putative target genes. Additional file 3:
**Primer sequences.**

## Abbreviations

bp: base pair
ChIP: chromatin immunoprecipitation
IL: interleukin
M-CSF: macrophage colony-stimulating factor
miRNA: microRNA
MO: monocyte
NF-κB: nuclear factor-kappa B
OC: osteoclast
PBMC: peripheral blood mononuclear cell
qRT-PCR: quantitative RT-PCR
RANKL: receptor activator of nuclear factor kappa-B ligand
SATM: sodium aurothiomalate
siRNA: small interfering RNA
TSS: transcription start site
UTR: untranslated region
