# Supplementary figures and images for: NF-κB-direct activation of microRNAs with repressive effects on monocyte-specific genes is critical for osteoclast differentiation
Source: Genome Biol. 2015 Jan 5;16(1):2. doi: 10.1186/s13059-014-0561-5 (PMC4290566; doi:10.1186/s13059-014-0561-5)

**A**

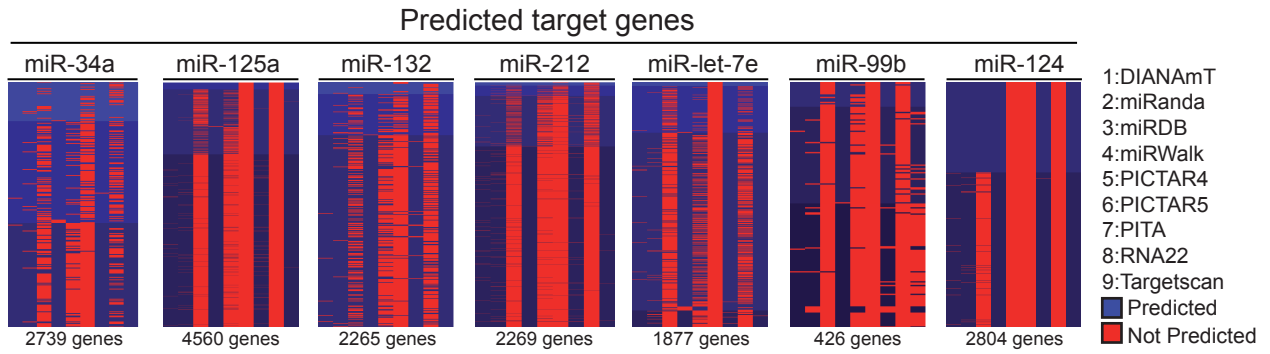**B**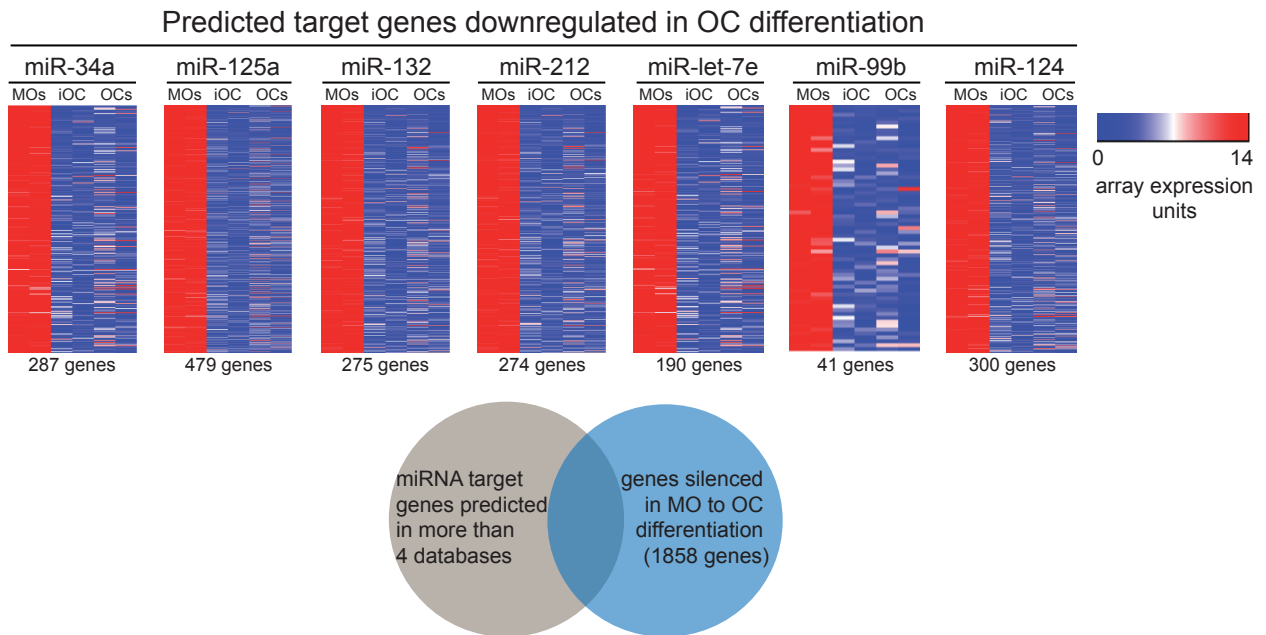

**C**

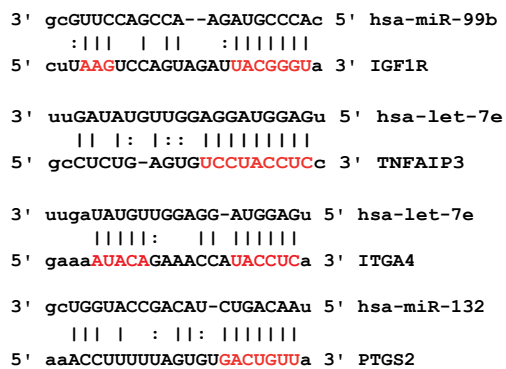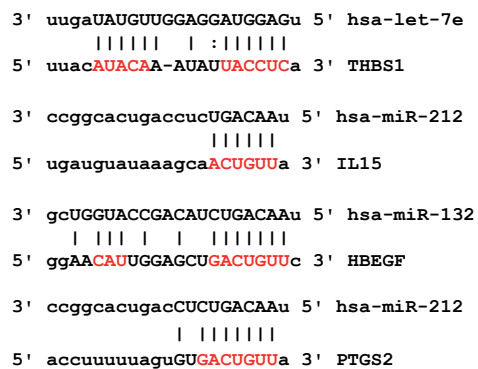

Supplement: Additional file 2: — (A) Heatmaps corresponding to putative targets for all miRNAs within the miR-99b/125a/let7e and miR-132/212 clusters using miRWalk. For a given prediction database (DIANAmT, miRanda, miRDB, miRWalk, PICTAR4, PICTAR5, PITA, RNA22, TargetScan) red corresponds to a positive match and blue indicates that it is not predicted. Only those putative targets predicted with at least four algorithms were used. (B) Overlap between the analysis with miRWAlk and expression data [32], using those genes that are downregulated in OC differentiation at least 0.5-fold. (C) Schematic representations of the pairing between different miRNAs and the 3′ UTR of different putative target genes. [file 13059_2014_561_MOESM2_ESM.pdf]
